# A 3D geological model of a structurally complex Alpine region as a basis for interdisciplinary research

**DOI:** 10.1038/sdata.2018.238

**Published:** 2018-10-30

**Authors:** James M. Thornton, Gregoire Mariethoz, Philip Brunner

**Affiliations:** 1 Centre for Hydrogeology and Geothermics, University of Neuchâtel, Rue Emile-Argand 11, 2000 Neuchâtel, Switzerland; 2 Institute of Earth Surface Dynamics, University of Lausanne, 1015 Lausanne, Switzerland

**Keywords:** Hydrology, Hydrogeology

## Abstract

Certain applications, such as understanding the influence of bedrock geology on hydrology in complex mountainous settings, demand 3D geological models that are detailed, high-resolution, accurate, and spatially-extensive. However, developing models with these characteristics remains challenging. Here, we present a dataset corresponding to a renowned tectonic entity in the Swiss Alps - the Nappe de Morcles - that does achieve these criteria. Locations of lithological interfaces and formation orientations were first extracted from existing sources. Then, using state-of-the-art algorithms, the interfaces were interpolated. Finally, an iterative process of evaluation and re-interpolation was undertaken. The geology was satisfactorily reproduced; modelled interfaces correspond well with the input data, and the estimated volumes seem plausible. Overall, 18 formations, including their associated secondary folds and selected faults, are represented at 10 m resolution. Numerous environmental investigations in the study area could benefit from the dataset; indeed, it is already informing integrated hydrological (snow/surface-water/groundwater) simulations. Our work demonstrates the potential that now exists to develop complex, high-quality geological models in support of contemporary Alpine research, augmenting traditional geological information in the process.

## Background & Summary

Three-dimensional (3D) geological models are digital representations of subsurface formations and their associated features. Recently, the appreciation of their utility to several disciplines has grown, and software tools enabling their construction have proliferated^1^. In earth sciences and engineering, they have, *inter alia*, contributed to the development of improved earthquake location catalogues^2^ and informed excavation and tunnelling projects^3,4^. They are also supporting ongoing radioactive waste storage site assessments^5^. In hydrogeology, meanwhile, they have facilitated groundwater resource estimates^6^, enabled the characterisation of karst aquifer geometries, flow pathways, and catchment areas^7–10^, and provided a basis for numerical modelling related to geothermal energy prospection^11^.

In tectonically and topographically complex sedimentary settings like the European Alps, 3D models must be generally detailed, high-resolution, and accurate in order to be suitable for their intended application(s). The term detailed refers to the representation of certain characteristic features which, here, would include folds, faults, and spatially-variable formation thicknesses. Developing a model with high spatial resolution, meanwhile, might involve employing a fine (e.g. cell size ≤ 10 m) Digital Terrain Model (DTM) to define the topographic surface, and/or using a sufficient density of georeferenced points to closely replicate the shapes of observed geological features. When exporting a model onto a grid or mesh, care must be exercised to ensure that the resolution is commensurate with the modelled features, especially thin layers or complex shapes. Finally, a model can be considered accurate if the estimated formations and associated features are close to their true positions (although the true positions may be impossible to establish perfectly).

Approximately 30% of the Alps are composed of carbonate rocks, the majority of which are karstified (i.e. discrete conduit networks have developed via dissolution)^12^. However, these rocks are not uniform in their chemico-mineralogical composition, and hence their degree of karstification. Moreover, they are commonly interspersed with lower permeability layers, such as marls and shales. The entire sequences have been folded, fractured, and faulted into complex geometrical arrangements by tectonic forces. Since well-karstified limestones are several orders of magnitude more permeable than marls and shales, the contrasts in hydraulic conductivity within these sequences can be considerable. Where so, the stratigraphic geometry exerts a profound influence on groundwater flow patterns^7^. For example, in a karstified limestone aquifer overlying a marly aquiclude, flow would typically be concentrated just above the interface, its direction corresponding to that of the maximum dip, i.e. flow would be broadly parallel to the strata (and so highly anisotropic). It follows that in folded settings, anticlines – assuming normal orientation – typically act as regional groundwater divides, with synclines conversely representing locations of accumulation^13^. Faults can also have a notable influence^14^; on one hand they may act as preferential pathway permitting flow across the strata, including enabling formations that would otherwise be considered aquicludes to be bypassed, but on the other, their offsets can disconnect aquifers.

As such, considering 3D geology is crucial when conceptualising and seeking to simulate groundwater flow in these environments. 3D geological models are considerably more powerful with respect to the development, visualisation, and communication of geological understanding than traditional 2D maps and cross-sections. They also provide a direct foundation for subsequent (3D) numerical flow modelling. However, for applications in topographically complex and potentially karstified limestone terrain, geological models must meet several criteria. Firstly, subsurface features that can affect flow must be accurately characterised. Secondly, to provide a realistic overall depiction, the topographic surface must be represented at high resolution. Finally, models must be spatially extensive enough to capture any proven or hypothesised subsurface connections; such connections can function over distances of up to several kilometres, and are capable of importing or exporting water across topographical boundaries.

Despite the improving capabilities of 3D modelling software and a large body of existing geological data pertaining to the Alps, these combined requirements (for geological models to be detailed, high-resolution, accurate, and spatially-extensive) continue to represent substantial technical and computational challenges to model development. It is therefore unsurprising that, irrespective of intended application, few such geological models exist in the Alps; those that do are generally very large in scale, and therefore limited in detail^15–17^, although there are exceptions in this regard^18^. Furthermore, the resultant datasets themselves are rarely made available as to the broad, interdisciplinary community who could potentially benefit from them.

Focussing on hydrogeology specifically, it may be noted that flow modelling tools which are capable of incorporating 3D geological information are increasingly widespread. Consequently, research into the interactions between geology and hydrology in Alpine regions is arguably now more limited by a lack of appropriate, accessible geological models than by flow simulation code capabilities. For instance, predicting the impacts of anthropogenic climate change on mountain streamflow regimes is an important and frequently undertaken task in hydrology^19–23^. However, (at least partly) due to a lack of explicit 3D geological information, such studies typically employ conceptual, box-type hydrological models like HBV^24^ and PREVAH^25^. These models have highly simplified structures and lack physically meaningful parameters, leaving them heavily reliant on calibration to reproduce historical observations. Indeed, they are commonly calibrated solely against stream discharge at the catchment outlet, despite multi-objective calibration and evaluation approaches – particularly those which consider spatially-distributed information – often being advocated^26–29^. Consequently, although it may be possible for such models to satisfactorily reproduce historical observations, they may be doing so for the “wrong reasons”^30^. Such a situation would compromise the robustness of any subsequent predictions, especially should forcing conditions exceed the range of the calibration dataset.

The representation of groundwater processes in box-type models is particularly concerning. Essentially, they contain at best only implicit information on the spatial distribution of subsurface properties. In many conceptualisations, fluxes between a soil-water reservoir and a groundwater reservoir are simply estimated as a calibrated function of the amount of water in the soil reservoir^31^. Even in spatially-distributed models, the subsurface is rarely discretised vertically, and lateral groundwater movement is generally unaccounted for. Consequently, any process understanding and predictions derived are in danger of overlooking site-specific geological influences. Even the user guide of the more physically-based and otherwise comprehensive model WaSiM recommends that coupling with an external groundwater model be undertaken wherever groundwater is expected to play an important role^32^.

Several reasons exist as to why groundwater can indeed be expected to play an important role in mountainous environments. Firstly, and most obviously, large hydraulic gradients exist. Secondly, as a result of the very tectonic processes that led to mountain formation, pronounced topography and geological complexity go hand in hand. Thirdly, it is known that this complexity in bedrock geology can strongly influence groundwater processes (and by extension overall catchment function) not only in calcareous settings, as discussed above, but elsewhere too^33–36^. Taken together, and alongside the fact that temperate mountain regions presently hold great hydrological importance for adjacent populations (primarily as a result of the orographic enhancement of precipitation and the storage and delayed release of water stored as snow and ice on seasonal and longer timescales)^37^, these points cause one to question whether the routinely made simplifications are appropriate.

Moreover, as a result of ongoing anthropogenic climate change, mountain hydrology research is becoming increasingly pressing. Two key components of such systems, the snowpack and glaciers, are already demonstrating pronounced sensitivity^38–40^. Accordingly, concerns about future water resources, especially during dry summer and autumn periods, are increasing^41,42^. Groundwater, vegetation, and permafrost will also respond to climate change, but may do so in a more subtle fashion, involving various interactions and feedbacks with other system components^43^. Predicting the overall changes in the quantity and timing of downstream discharge thus requires more advanced hydrological models. Even prior to that, however, our fundamental knowledge of how and to what extent high-elevation aquifers are recharged, as well as how they transport and discharge water to maintain stream baseflows and spring discharges, must urgently be improved^44,45^.

Integrated hydrological models like ParFlow-CLM^46^, HydroGeoSphere (HGS)^47^, MIKE SHE^48^, or CATHY^49^ may be useful in both regards. Such models generally solve equations for 2D surface and 3D subsurface (both saturated and vadose zones) simultaneously and, crucially, can explicitly represent 3D variability in hydraulic properties defined, for instance, according to a 3D geological model. They also represent most other pertinent elements of the water cycle (including snow accumulation and melt), and simulate the interactions between them, in a coherent, spatially explicit, and transient fashion. In HGS, for instance, several options also exist with respect to karstified formations; the subsurface can either be treated as an equivalent porous media at the elemental scale, as having dual permeability or porosity, and/or as being discretely fractured. All this is possible whilst simultaneously simulating; i) surface flows (important for flood risk and sediment transport), ii) interactions between soil moisture/groundwater, vegetation characteristics, and evapotranspiration, and iii) snow processes. Theoretically, these capabilities leave integrated models uniquely placed to quantify the physical relationships between climate, geology, hydrology, vegetation, and snow in mountainous environments. However, they have found few applications in steep, geologically complex terrain to date. The limited availability of data with which they might be parameterised, calibrated, and evaluated – including 3D geological information – may be posited as an important contributory factor.

In this context, and as part of an interdisciplinary project seeking to improve predictions of Alpine water availability and vegetation species distributions (http://wp.unil.ch/integralp), a novel dataset characterising a section of a well-studied nappe fold in the Swiss Alps is presented. No 3D model of this complex region previously existed, and it was unclear at the outset whether developing an appropriate model was even feasible. Alongside various other datasets, the resultant dataset is currently informing catchment scale, integrated hydrological modelling efforts (to be presented in subsequent publications). Various other ongoing or future interdisciplinary environmental investigations could also benefit from the development.

## Methods

### Study area

#### Geological context

**Figure 1:**
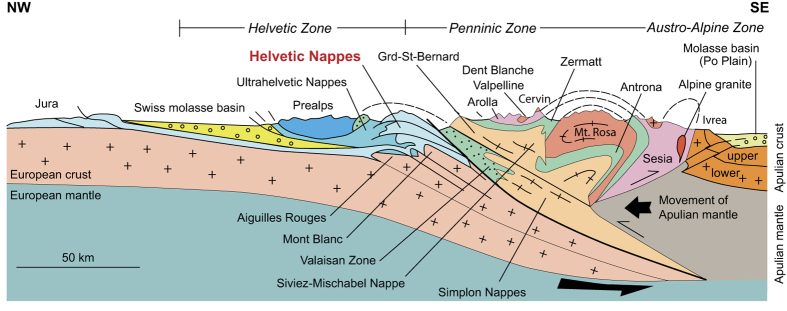
The situation of the Helvetic Nappes in a profile through the European Alps. The Nappe de Morcles, which is the focus of this work, is the lowermost of the three nappes (Source: modified after Renard *et al.*^82^ © Dunod, 2015).

The Nappe de Morcles (western Swiss Alps) is a world-renowned example of a tectonic nappe fold, having an amplitude exceeding 10 km and a prominent inverse limb whose stratigraphy is completely reversed. It is the lowest of several such tectonic entities that strike to the SW-NE and together comprise the Helvetic Nappes (Fig. 1). The nappe sits above a region of autochthonous and parautochthonous material which, in turn, overlies the crystalline Aiguilles Rouges massif. It is composed primarily of calcareous shelf sediments (limestones, marls, shales, and sandstones) of Jurrassic to Paleogene age.

#### General characteristics of the focus area

**Figure 2:**
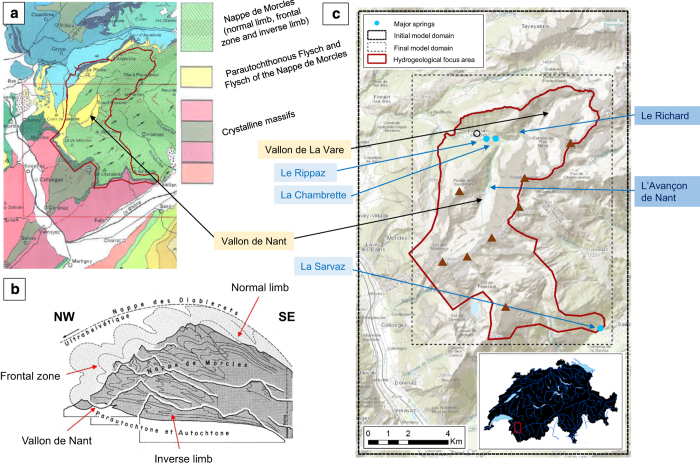
The study area. (**a**) Tectonic sketch indicating the general geological situation (reproduced from Badoux^53^, non-digital version, © Source: Swiss Federal Office of Topography, 1971). (**b**) Illustrative cross-section through the Nappe de Morcles showing its pre-erosion structure, including secondary folds, and the present topographic arrangement (reproduced from Badoux *et al.*^53^, © Source: Swiss Federal Office of Topography, 1971), and c) The “original” and “final” geological model domains, major springs locations and the area of hydrogeological interest (the “focus area”) (original figure). In (**c**), selected peaks are marked (brown triangles); starting from the north and proceeding clockwise, these are: Tête à Pierre Grept (2,904 m), Grand Muveran (3,051 m), Petit Muveran (2,810 m), Grand Chavalard (2,899 m), Dent Favre (2,917 m), Grand Dent de Morcles (2,969 m), Petit Dent de Morcles (2, 929 m) and Point des Savolaires (2, 294 m). The village of Les Plans-sur-Bex (hollow black circle) is also marked.

At the outset of the wider project, two adjoining valleys in – the Vallon de Nant and the Vallon de la Vare – were identified as the focus for subsequent hydrological model development. The geological model domain was therefore centred on this area. These valleys lie within the north-western section of the Nappe de Morcles (Fig. 2). More specifically, the Vallon de Nant has been eroded from the inverse zone of the nappe, whilst the Vallon de La Vare lies in the frontal zone. For reasons that shall be explained shortly, the focus area was extended to the southeast to include La Sarvaz spring.

The elevation range within the focus area is considerable (~2,500 m), and precipitation abundant (annual average ~1600 mm·yr^-1^ in the lowest reaches, increasing with elevation). Low winter temperatures result in a significant proportion of the annual precipitation falling as snow^62^, and small glaciers are able to persist at relatively low elevations in the uppermost parts. Considerable diversity is encountered with respect to vegetation, geomorphology, and hydrology. The area has remained practically untouched by anthropogenic activity; indeed, the Vallon de Nant has been a designated natural reserve since 1969. Overall, the area represents an ideal “natural laboratory” for research across the environmental sciences^63^.

**Figure 3:**
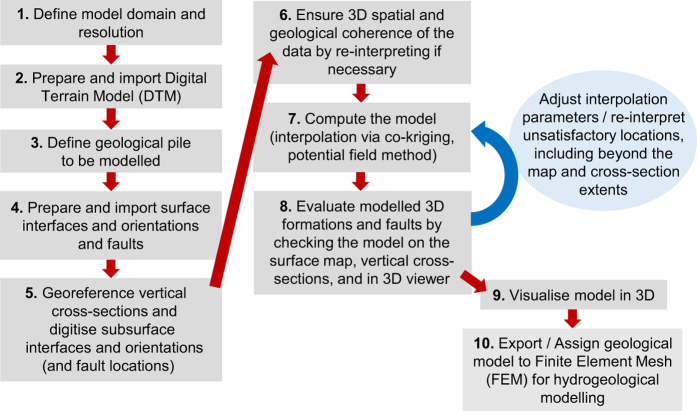
The workflow followed to develop the 3D geological model. Steps 7 and 8 involved an iterative process of: i) comparing the model to the input data on both the surface and vertical sections, ii) adjusting the interpolation parameters and/or the input data density, iii) re-computing the model, and iv) re-comparing with the input data.

The first stage of the geological model development involved establishing the spatial domains (see Fig. 3). Formations belonging to the other Helvetic Nappes or the Ultrahelvetic zone were excluded from the model to keep the degree of complexity manageable.

#### Stratigraphy and initial hydrogeological inferences

A sketch of the stratigraphy in the area of the Vallon de Nant, modified after Badoux^59^ (Supplementary Figure 1), provides a useful introduction to the regional geology. (Note that this diagram does not include all the formations that were eventually modelled; for that, consult Table 2). Having said that, the 1:25,000 scale geological maps of “Morcles”^53^ and “Les Diablerets”^56^ represented the primary sources of detailed geological information. The lithological descriptions provided in the accompanying explanatory notes enabled the likely hydrogeological importance of certain formations to be promptly identified. Reviewing previous studies conducted on neighbouring or nearby tectonic units where equivalent formations are encountered^12,64^ further elucidated the probable hydrostratigraphy.

In this way, it was possible to establish, for instance, that the massive Urgonian limestone is likely to represent an important karst aquifer(s); this formation is renowned for its purity at other sites, and it hosts a major aquifer in the overlying Nappe de Diableters^64^. The Malm and Valanginian limestones are also likely aquifers^14^. The more siliceous Hauterivian limestone should still be permeable, but probably offers more resistance to flow^65^. In contrast, the Berremian marl is likely to act as a regional aquiclude, as has been reported elsewhere in the Hevetic zone^12^. However, the thinness of this unit in our study region (only ~ 30 m in the region of the Vallon de Nant, according to Supplementary Figure 1), and its inability to prevent a hydrological connection with La Chambrettes^65^, mean that its effectiveness as an aquiclude at our site is not guaranteed. The “top” of nappe (i.e. the “bottom” in areas with inverted stratigraphy) is comprised of a thick, clayey Oligocence flysch that is expected to have very low permeability on a regional scale. This brief overview merely seeks to highlight some potentially important formations, and should not be considered exhaustive.

Few hydrological or hydrogeological investigations have previously been conducted in the study area. The karstic source of La Chambrette, which emerges above the northern bank of L’Avançon de Nant near the village of Les Plans-sur-Bex, was, however, the subject of an early artificial tracer test^65^. This experiment demonstrated the existence of a hydrological connection between the closed basin of “La Varre” and the spring. Since these two locations are separated by a formation (the Lower Barremian, as mentioned above) that should be rather impermeable according to its lithology, it was proposed that a fault of some description must enable flow to traverse it. Today, the spring is exploited by Romande Energie SA. Another spring, Le Rippaz, is no longer karstic by the time it emerges rather diffusely from Quaternary moraines, a little downstream from La Chambrette on the opposite (southern) bank of L’Avançon. Currently, it is being developed to augment the water supply of a neighbouring commune^66,67^. An associated tracer test confirmed that the formations of the Lower Cretaceous near Pointe des Savolaires constitute the main karstic aquifer. This system could thus be thought of as a chain of aquifers – first karstic and then unconsolidated gravel – which together dampen the variability of spring discharge to snowmelt and rainfall inputs. Finally, as mentioned earlier, the focus area was extended several kilometres to the southeast to include La Sarvaz; another, higher discharge karstic spring that emerges near the village of Saillon, Valais. This decision was taken because, according to our initial understanding of the geological structure and likely favourable hydraulic properties – specifically of the Malm – some the precipitation falling on the southern and eastern ridges of the Vallon de Nant may ultimately drain to this location, rather than via L’Avançon de Nant. Additionally, the discharge of La Sarvaz has been monitored for several years, providing observations that could prove useful during hydrological model development.

One aspect of the regional geology that remains somewhat unclear is the extent to which the various theoretically karstifiable formations are actually karstified at this site. The only known speleological exploration in the region returned an inventory of only six small caves^68^, although this exploration was neither systematic nor exhaustive. The walls of the Urgonian limestone near Pointe des Savolaires were reported to be the only limestones pure enough to contain cavities of speleological interest^70^. However, while the presence of caves is an unambiguous indicator of karstification, the inverse is not true; an absence (or non-discovery) of explorable caves certainly does not necessarily indicate an absence of hydrologically meaningful karstification, since conduits much smaller than humanly accessible can still transport significant volumes of water extremely rapidly. Nevertheless, it should be remembered that flowing water is required for conduit development; if a theoretically karstifiable limestone has been disconnected from recharge or circulation by low permeability layers, it may remain little karstified.

Although the conclusions of this speleological prospection broadly concord with our hydrogeological expectations drawn from the lithological descriptions, they do highlight the issue of chemio-mineralogical purity, and more specifically the possibility that the degree of karstification in our study area may not be as high as elsewhere in the Helevetic zone. In any case, a benefit of our approach – of only estimating formation geometries initially – is that in contrast to the methodology followed by other authors^8,10^, there is no need to definitively categorise each formation as being either an aquifer or aquiclude initially; reality is certainly not this binary. Rather, it is our intention to vary and hopefully constrain parameters representing the hydraulic properties of the various formations during subsequent numerical model calibration.

Finally, aquifers are of course not confined to bedrock formations. Glacio-fluvial and other sediments (e.g. talus cones) fill the valley floors. It is expected that the recharge of these units (where permeable) is dominated by spring snowmelt, with drainage subsequently taking place over the course of the late spring, summer, and autumn via a number of springs and ephemeral “seeps”. The geological model presented herein does not include these unconsolidated formations, focussing instead on the more voluminous bedrock. That said, geoelectrical surveys will be undertaken shortly with a view to representing the geometry and heterogeneity of unconsolidated material properties in the integrated hydrological model.

#### Surface hydrology and its association with bedrock geology

The surface hydrology of the Vallon de Nant is characterised by the eponymous Nants; the torrents that course down its steep slopes. They are located principally to the south and east, and have a highly variable flow that responds rapidly to rainfall and snow and ice melt. The most important are the Nant des Têtes and the Torrent des Martinets, which together constitute the majority of the discharge of L’Avançon. The torrents are also responsible for a great deal of sediment transport; these deposits accumulate to form a large alluvial fan in the middle section of the valley.

The annual hydrological regime of L’Avançon de Nant may be classified as *nivo-glacial*^69^; that is to say, it has a mixed snow and ice-dominated response with a discharge peak in early summer corresponding to melt of the snowpack, variable discharges from one year to another, diurnal cycles superimposed on the hydrograph due to ice melt, and groundwater contributions maintaining baseflow.

Surface water is noticeably scarcer in the upper part of the Vallon de La Vare than the Vallon de Nant, although a small stream – Le Richard – does develop and joins L’Avançon downstream of Pont de Nant. This contrast between the two valleys can probably be explained by differences in their position in the nappe. Specifically, the Vallon de Nant is underlain by low-permeability flysch, which limits groundwater exportation and permits the existence of a relatively long section of permanent stream (it is only ephemeral in the upper section in dry, cold autumn and winter periods, when the groundwater level in this upper section has fallen such that it can no longer contribute to streamflow). A clayey layer of glacial till at some intermediate depth between the surface and bedrock interface may also contribute to this behaviour, although this hypothesis remains to be tested by geophysics. In contrast, the Vallon de La Vare lies in the inverse zone, and thus the impermeable flysch is oriented approximately vertically away to the north, near L’Argentine. Hence, the bedrock beneath the valley floor is composed of the more permeable limestones of the Lower Cretaceous. One aim of the 3D model is to help visualise and better understood such influences. Additionally, having two somewhat geologically contrasting catchments adjacent to one another provides the opportunity to explore the specific influences of geology on hydrology whilst other factors, e.g. climate, remain fairly constant.

### Input data

A wealth of geological information pertaining to the European Alps exists, having been developed over decades of dedicated study by committed regional experts. Presently, this information exists primarily in the form of two-dimensional (2D) maps and cross-sectional diagrams. Despite observational advances in most other fields of the geosciences, the field mapping techniques and concepts underpinning the production of such structural geology datasets have changed little in over a century^70^. As such, their quality remains similar to more contemporary outputs. Moreover, the prevalence of observable features such as stratigraphic interfaces, faults, and folds mean that such datasets are typically more accurate in mountainous regions than in settings where the bedrock is more obscured by unconsolidated deposits^18^. Indeed, the deep incision made by the Vallon de Nant into the inverse limb of the nappe can be thought of as a kind of “window” into its interior − a visible cross-section. Certainly, the availability of appropriate geological data should rarely be a limiting factor for the development of 3D models in the Alps, although the arguable under-exploitation of the existing body of information means it might have been hitherto.

As already mentioned, thanks to its reputation as a classic example of a first-order nappe and intriguing attendant complexity, the structural geology of our study region has a long history of being studied and mapped. At this juncture, it is worth briefly highlighting the effort that the production of the maps, cross-sections, and associated explanatory notes that comprise the current Geological Atlas of Switzerland in this region entailed. In the introductory remarks to his illustrated text *Tectonics of the Morcles Nappe between Rhone and Lizerne*, Badoux states that the work undertaken prior to the publication of the second edition maps took 8 years!^54^ By this time, under the tutelage of Lugeon, he had already developed a great passion for, and expertise on, the geology of the Vaud Alps. These maps remain the highest resolution, most current geological dataset of the Swiss Confederation in this region.

As Table 1 indicates, input data for our model were derived from three primary sources: i) a Digital Terrain Model (DTM), ii) surface geological maps, and iii) vertical geological cross-sectional diagrams.

### Workflow

Having gained an appreciation of the study area and identified and sourced appropriate input data pertaining to it, several sequential steps were followed in order to develop the geological model (Fig. 3). In summary, data extracted from existing geological maps and vertical cross-sections were compiled along with a digital terrain model (DTM) in the GeoModeller software environment^71^. GeoModeller is a commercial platform developed by the BRGM (French Geological Survey) and Intrepid Geophysics; for further information, see Calcagno *et al.*^72^ and Guillen *et al.*^73^. It facilitates the estimation of continuous geological models that respect all available data indicating the locations of interfaces between different lithological formations, the spatial orientations of these formations, and any faults present in the domain. Certain geological rules are also taken into account. The following sections describe each phase in more detail.

#### Defining the model domain and resolution

Studying the regional geology and hydrogeology enabled the initial and final domains for the geological model development to be established (Fig. 2). The initial model domain was slightly larger than the final one to ensure that all data that could potentially inform the geological model in the smaller focus area were included in the estimation. In light of the complex topography and our prior knowledge of the presence of geometrically complex and thin units, it was decided that the model development should proceed at a resolution of 10 m.

#### Preparing and importing the Digital Terrain Model (DTM)

The swissALTI^3D^ digital terrain model (DTM) is a raster dataset with a horizontal cell resolution of 2 m. It represents the land surface without vegetation or buildings. For further information, see: https://shop.swisstopo.admin.ch/en/products/height_models/alti3D.

The vertical uncertainties associated with the product, quoted in Table 1, are more than low enough for the application at hand.

For the purpose of model development, the dataset was resampled to 10 m. This resampling served to smooth out high-frequency noise (i.e. small-scale topographic features) and reduce the computational burden. The resultant dataset was imported into GeoModeller (Supplementary Figure 2). It forms the upper surface of the geological model.

### Defining the geological pile to be modelled

The term geological pile refers to the sequence of lithological formations to be modelled. Since the domain extends over two separate geological map sheets, each having a slightly different formation classification scheme, some reconciliation was required to arrive at a single sequence. In the end, the sequence modelled was extremely similar to that of the Morcles map sheet. Given the potential multi-disciplinary applications of the dataset, maintaining a classification that closely resembled that of the map legends was considered preferable to grouping any formations *a priori*. Of course, should further simplification be required for a particular end use, the formations represented in the output dataset can be grouped later.

#### Preparing and importing surface formation interface data, orientation data, and faults

**Figure 4:**
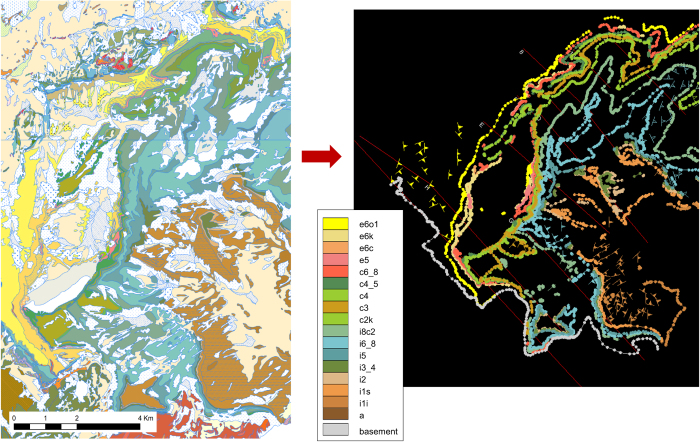
Definition of surface geological input data. Georeferenced point features (formation interface locations and marked structural data) extracted from the surface map (left, Badoux *et al.*^53^, Badoux and Gabus^56^, Source: © Swiss Federal Office of Topography, 1971, 1991) and imported into the GeoModeller interface (right). The legend refers to the coding of the formations of the stratigraphic pile. Circles indicate interface points, and arrows indicate orientations. Note that since the DTM had already been imported at this juncture, these points were in fact located in 3D and not only 2D space (i.e. they could be associated with a z-value).

Two types of surficial geological data from the relevant map sheets^53,56^ were obtained in digital format (Fig. 4). These were: i) polylines formed by joined points indicating the locations of the interfaces between the formations of the geological pile which outcrop at the surface (also known as “contacts”), and ii) point features describing the orientation of these formations at certain locations on the surface (also known as “structural data”).

Where necessary, interface polylines were reattributed to match the formations defined in the geological pile. Whilst taking care to maintain their shapes, they were also simplified to reduce the number of vertices. This spatial data processing was conducted using the open source software QGIS. The processed polylines were then imported into GeoModeller. Given the complexity and number of interface shapes in the study region, this approach was more efficient than the more common practice of importing a pre-georeferenced image of a geological map into GeoModeller and then manually digitising the surface formation interfaces in that software environment. In locations where it was clear that a given boundary was continuous beneath the Quaternary cover (and hence was not actually defined) but the location of the boundary could be easily estimated, additional points were inserted. In certain other locations, bedrock interfaces completely obscured by the surficial cover could not be estimated. The consequent data gaps represent a source of uncertainty in the final model. (The model proposes continuous boundaries as a result of the interpolation).

The orientation points were attributed to formations of the geological pile by means of spatial intersection. Some surface orientation data points that fell between vertical cross-sections in the heavily folded frontal zone had to be discarded because at these locations, the polarities/younging directions (i.e. whether normal or overturned) could not be determined with confidence.

Faults with more limited offsets, which are not represented, could still be responsible for preferential hydrological flow pathways; an effect that could be represented in any subsequent hydrogeological modelling by prescribing discrete fractures, or by treating the corresponding volumes as having dual permeability.

#### Georeferencing vertical cross-sections, digitising subsurface interfaces and orientations

**Figure 5:**
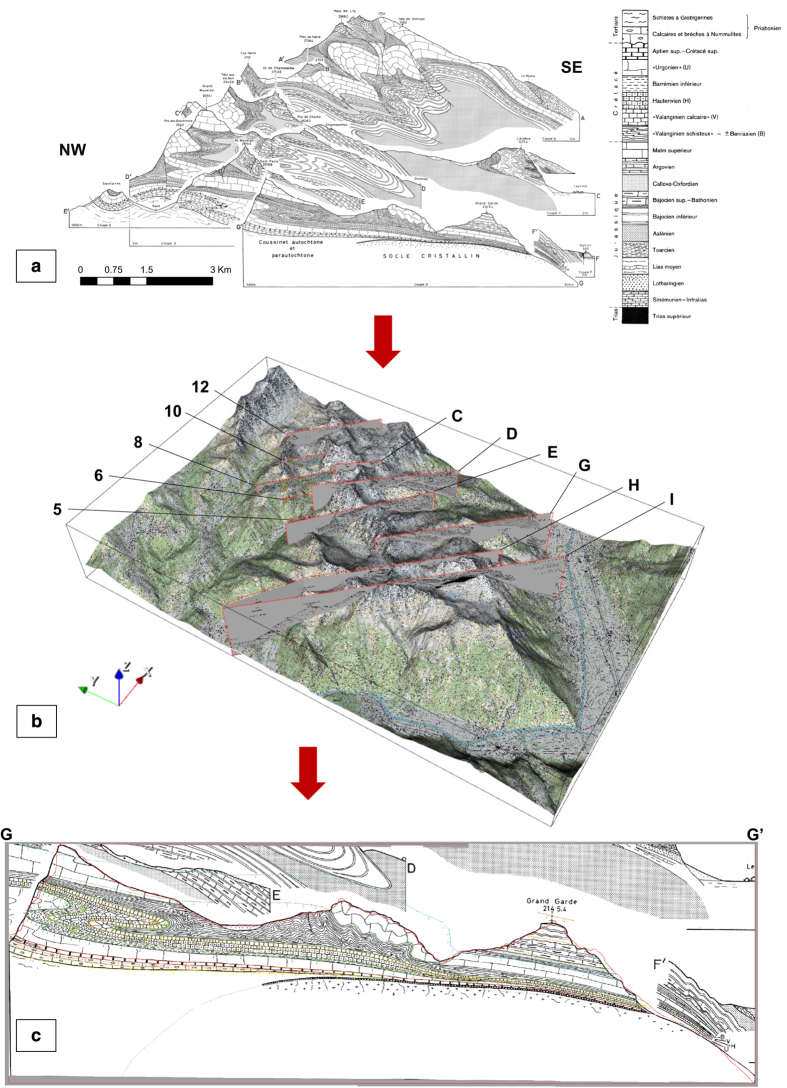
Steps undertaken to process the subsurface geological data. (**a**) Incoming vertical cross-sections. Note that only C-I (i.e. those from the Morcles map sheet) are shown in this panel (Badoux *et al.*^53^, Source: © Swiss Federal Office of Topography, 1971), although Sections 2-12 (from the Diablerets map sheet; Badoux and Gabus^56^, Source: © Swiss Federal Office of Topography, 1991) were also used in the model development, (**b**) All 11 cross sections, having been georeferenced, visualised in the 3D viewer of GeoModeller, and (**c**) An example of a georeferenced cross section (GG’) visualised in the 2D viewer of GeoModeller, with subsurface interface and orientation data points digitised according to the locations and dips of the various interfaces shown (coloured lines and arrows, respectively). Once these sections were viewed, it was also ensured that the topographic surface illustrated on the diagram closely matched that derived from the DTM along the same profile (red line); a close match, as seen in this example, indicates sound georeferencing. Furthermore, since the DTM has been developed using modern technology (LiDAR and photogrammetry) and is therefore of high quality, a close match also indicates an accurate representation of the topographic surface in the original figure.

The “stacked” vertical cross-sections^53,56^ (Fig. 5a) provide interpretations of the subsurface structures. Including such information in the estimation of model was crucial because the non-stationarity of the domain, which arises due to the pronounced folding and faulting, precludes the straightforward extrapolation of surface observations into the subsurface.

**Figure 6:**
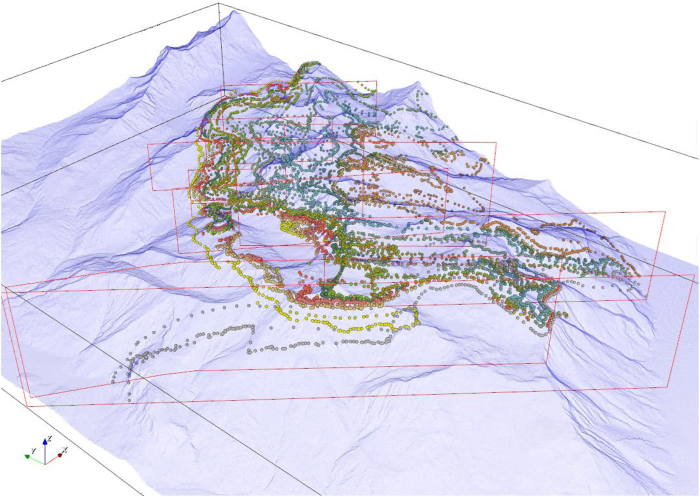
All digitised geological formation interfaces, derived from both the surface maps and vertical cross-sections, visualised in 3D with a semi-transparent topographic surface. To avoid cluttering, only interface data points are shown, although orientations were also visualised and checked in the same fashion. Here, the view is towards the north-east.

Prior to being imported into GeoModeller, the diagrams were cropped and the cross-section start and end locations georeferenced (Fig. 5b). For each cross-section, the correspondence between the topographic surface profiles in the GeoModeller environment (i.e. the profile of the DTM between the georeferenced cross-section start and end points) and the representation of the topography on the georeferenced diagram was assessed (see the red line in Fig. 5c). The close matches observed gave us confidence that both the georeferencing of the cross-section diagrams and the original representation of the topography along the sections were satisfactory.Fig. 6

The lengthy task of manually digitising the subsurface formation interfaces illustrated on the cross-sections, and their associated orientations, was then undertaken. In this process, the interface surfaces were assumed orthogonal to the section plane, i.e. the dip direction is parallel to the section, with dip angles estimated at regular intervals along each interface according to the angles formed with the horizontal plane. Due to the plunge of the axis of the main nappe structure, the subsurface dip and dip directions resulting from this assumption may not be entirely correct in all instances. However, the approach taken was the only practical way in which some subsurface orientation data could be included, which in turn was absolutely necessary to successfully model the region. This assumption is not expected to have any implications for the utility of the model for hydrological and other environmental applications.

In total, 11 cross-sections distributed throughout the domain contributed to the model.

#### Computing the model

In contrast to more traditional “explicit” or “surface-based” approaches, which essentially involve users generating geological surfaces or volumes based directly on the available data, GeoModeller takes an “implicit approach” to model estimation. Such an approach relies on algorithms that integrate observed data with geological interpretation. The particular theory and algorithms that are employed have been comprehensively described and exemplified elsewhere^72,74,75^, but to summarise: formation interface and orientation data are co-kriged to produce a 3D scalar field, or potential field^76^. Equipotential iso-surfaces with certain reference values represent formation interfaces, whilst the gradients of the scalar function describe their orientations. Several potential fields can be combined in the same model to, for instance, reconstruct complex erosive and/or onlap relationships between geological series (which are simply groups of individual formations). Faults can be represented by inserting discontinuities into the potential field. Once computed, the surfaces or volumes may be visualised by means of a marching cube methodology^77^.

The key benefits of taking an implicit approach are that certain conditions of geological validity (e.g. the forbiddance of overlapping interfaces) are directly enforced^78^, and that interface contact and orientation data can be considered simultaneously in the estimation of a continuous model^79^. Another positive feature is that a given model can be updated relatively quickly following the addition of new data. In our case, this allowed several “competing” models to be generated efficiently using slightly different subsets of the data, alternative interpretations of the relationship between formations in the geological pile, and varied parameter values. Drawbacks of the implicit approach include its relatively high memory usage, and the limited opportunity that exists for users to manually adjust individual modelled surfaces to match their expectations or expert knowledge.

The first stage of computing the model involved testing how the relations between the series (which are simply groups of formations) of the geological pile might best be represented. According to the legend of the Morcles map, erosive relationships exist between the Jurassic and the Cretaceous series, and also between the Cretaceous and Tertiary series. In early iterations these series were indeed treated separately with “Erode” relations, such that one or more would take precedence and cut over the others (depending on which were defined as “Erode” and which remained “Onlap”, as well as their relative positions in the pile). However, this prevented data from the upper or lower formation of one series from constraining formations in the next series, leading to some cross-cutting situations that were deemed unrealistic. Because the interfaces of all formations seem to generally follow one another, with few if any instances of formations within the modelled series having been completely eroded, it was eventually decided to treat all formations as part of a single series.

The only further inputs necessary to compute a model are the values of three (isotropic) interpolation parameters: the range, the nugget effect of geological interface data, and the nugget effect of geological orientation data. The range represents the spherical distance beyond a given location at which data points will have no influence on the model. The nugget effect parameters represent the variance between the values of observed data points that is not explained by separation distance, but could rather reflect measurement error or stochasticity (i.e. “noise”). Here, it can be thought of as the mismatch permitted between model and data.

### Code availability

GeoModeller is a proprietary software owned by, and licensable from, Intrepid Geophysics (v.3.3.0 was used in this work). The approach GeoModeller takes has been thoroughly described in the peer-reviewed literature. Open source GIS tools (QGIS; https://qgis.org/en/site/) were applied for data preparation.

## Data Records

### Exporting the model

Once an acceptable model had been produced (see Technical Validation), the model was exported in a regular voxel format at both 10 m and 50 m resolution. In this format, to reduce data volumes, the position of each voxel is given implicitly. In principle, the voxel model can be used directly for hydrogeological modelling. However, this would be rather inefficient with respect to the number of nodes and elements required. More contemporary practice would be to separately generate a finite element mesh of variable resolution, which allows appropriate representation of important features whilst minimising the total number of nodes, and then assign geological codes to each element in this mesh according to the geological model via a spatial query. Following this, one could attribute (based on knowledge of the lithology, etc.) reasonable initial estimates of the values of parameters describing physical properties throughout the domain. Such meshes could be comprised of layered prisms or, as are beginning to emerge, fully-unstructured tetrahedra. Various possibilities exist for refining or optimising the arrangement of elements according to surface or subsurface features, although this topic lies beyond the scope of this work.

3D shapes were also exported for visualisation. More specifically, the formation interface surfaces may be loaded as polygonal meshes in ParaView^80^; an open source 3D visualisation software. In this environment, layers can be visualised or hidden at the user’s behest, and virtual interaction undertaken.

Finally, the final GeoModeller project was saved in its native format.

The model data (Data Citation 1) is provided in a generic voxel format at two different resolutions: 10 m and 50 m (regular cubic cells) (Generic voxel format, Data Citation 1). The lithological code is listed for each cell. For ease of processing, no refinement of the vertical resolution has been made near the interface with the topographic surface in either case. The 10 m resolution voxel model is also provided in a modified voxel format that can be loaded into the freely-available visualisation software SGeMS^81^ (SGeMS voxel format; also see Supplementary Figure 4). In addition, formation interface surface were prepared in a format that can be visualised in ParaView (Surfaces for ParaView, Data Citation 1). Finally, the entire GeoModeller project is provided in its native format; this can be viewed by readers with a GeoModeller licence (Native GeoModeller Project, Data Citation 1).

In the SGeMS format, the lithological formations are represented by the codes listed in Table 2.

## Technical Validation

The most relevant evaluation process was the visual comparison of how well lithological formation interfaces defined by the input data compared with the corresponding modelled interfaces – both on surface and on the vertical cross-sections. Accordingly, as part of the iterative computation and re-evaluation process, the adjustable parameters were systematically varied until a satisfactory balance between a smooth model and model that honoured the data was obtained. In practical terms, this was achieved by visually comparing the input data and the modelled interfaces on both surface and vertical sections. The final parameter values we arrived at are:

- Range: **2,000** m
- Nugget effect on geological interface data: **0.00001** (arbitrary unit of the potential field)
- Nugget effect on geological orientation data: **0.1** (arbitrary unit of the potential field)

**Figure 7:**
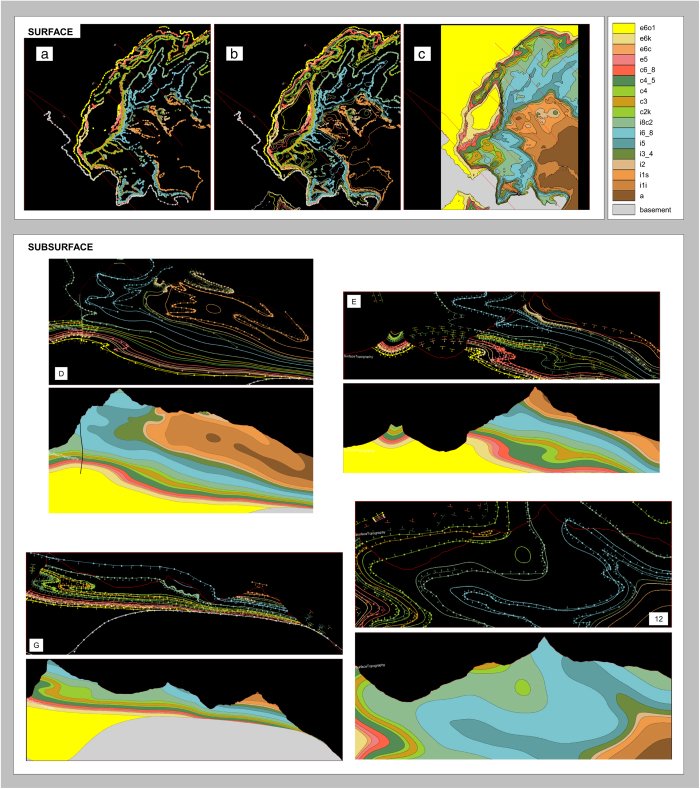
Comparison of input interface data points and modelled interfaces. The thin lines with circles and arrows correspond to the input data extracted from the surface maps (surface section), or digitised directly from the formation interfaces illustrated on the cross-section diagrams (vertical sections). Specifically, the circles represent interface data points and arrows represent orientation data points. Input orientation data points are not shown on the surface section in the interests of clarity. The thicker, continuous lines correspond to the modelled surfaces. For the surface, the data points alone (**a**) and then data points with the interpolated interfaces underneath (**b**) are shown separately. The final filled volumes are also shown for all sections (panel **c** shows this for the surface section). Vertical cross-section letters follow the convention of the original inputs. In Section D, one of the faults that has been modelled is visible. Two disconnected circles (spheres in 3D) are visible; these are model artefacts, and are discussed in due course.

Comparisons of the input data and final modelled interfaces on the surface section and four randomly selected vertical cross-sections are shown in Fig. 7. The close matches obtained indicate that the interpolation algorithm can reproduce the structures defined in the input data. If one then assumes that the original geological interpretation presented in the maps (i.e. the input data itself) is reasonable, one may suggest that the new 3D model is also geologically plausible. Taking slices through the domain between cross-section locations, and visualising the estimated volumes of individual formations with their corresponding input points, reinforced this assessment.

Although the number of input data points observations is high, given the inaccessibility of the subsurface, they are still unlikely to be sufficient to constrain a unique model^79^. For this reason, no data were explicitly withheld to provide independent evaluation data; keeping any observations aside would inevitably have had an adverse impact on the final model.

**Figure 8:**
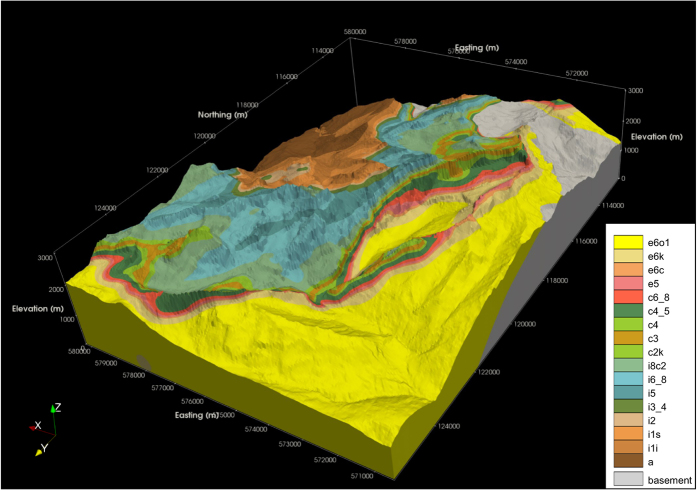
Illustration of the final coherent 3D model of bedrock geology, looking towards the south-west. This figure was produced in ParaView. Coordinates are given in the CH1903 / LV03 system. The formation codes correspond broadly to those of the legend of the 58 Morcles sheet^53^, although the geological pile was slightly modified to reconcile it with the Diablerets sheet. Some colours were slightly adjusted to increase visual impact. In the western part of the domain, the stratigraphy is overturned, i.e. geologically older units are found above younger ones. Quaternary cover is not included in the model. Formations of the Ultrahelvetic zone are not included in the model, and thus the presence of “e6o1” (bright yellow) in the extreme north-west of the figure is not reflective of the real geology in this region (as indicated in Fig. 2a). Similarly, the model is less well constrained in the extreme east of the domain (i.e. towards the centre of the nappe), which is away from the main area of interest.

The resultant dataset (Figure 8) represents 18 formations, including their associated folds and selected major faults, and covers a horizontal area of 9.6×13.4 km.

A further visual comparison was made between a panoramic sketch of the geology of the eastern side of the Vallon de Nant (“Planche II” of Badoux^54^) and a roughly equivalent view produced in the modelled environment, again with satisfactory results (Supplementary Figure 3).

The interpolation approach employed to develop the 3D model is based on co-kriging. For this reason, the density of data points may have some effect on the final model. Whilst the relatively high-density surface points were extracted directly from the (pre-digitised) map data, and were therefore rather treated as rather “fixed” (although the density of vertices on relatively straight surface interfaces was increased so these features were not unduly de-weighted), the subsurface points had to be digitised manually from the cross-sectional diagrams. The density at which these points were inserted was at the discretion of the model developer, and was therefore somewhat arbitrary. This inevitably introduced a degree of subjectivity into the modelling process. On the other hand, it also provided a degree of flexibly to vary the extent to which data constrain the model regionally. For instance, where uncertainty in the position of an interface was considered lower (e.g. near an observable interface at the surface), the density of points could be increased. Furthermore, where the precise location of the interface was unknown in a particular region but the orientations could be inferred from adjacent layers, subsurface orientation data points could be inserted independently of interface data. Finally, as mentioned above, some additional interface points were even inserted onto the surface section where it was clear that a given boundary was continuous but was simply not marked on the map as a result of being obscured by Quaternary deposits. Accounting for these aspects, rather than simply computing the model based only on the available data, enabled us to produce a final model that both honoured the data and could be deemed geologically plausible.

The resultant model is deterministic. In regard to this, it may be noted firstly that the number of outcrops present in our highly eroded, mountainous study area mean that the geological structures are relatively easy to observe. Therefore, although the area is geologically complex, and the existing data (which formed our inputs) are naturally associated with some uncertainty (the vertical cross-sections naturally more than the surface data), this data uncertainty is arguably less pronounced than in other geological settings where bedrock outcrops are less conspicuous. The series of vertical cross-sections is also very dense. As such, it seems unlikely that attempting to refine the field data or geological interpretation, as expressed in the existing surface mapping or vertical cross-sections, would yield major improvements in the output.

Nevertheless, in future work, it would be possible to assess the uncertainty associated with the deterministic geometrical representation to be assessed by generating multiple realisations of the model. This could be achieved by perturbing the parameter values within a probabilistic framework. That said, it may be suggested that the high data density leaves little scope for model variability during the interpolation stage. Furthermore, before embarking on such a task, the relative magnitudes of various uncertainties that could arise throughout a wider modelling chain should be contemplated. For example, in our hydrogeological application, the uncertainty in the hydraulic conductivities of the various formations is likely to be much more substantial that the uncertainty in their geometrical structures. Exploring the capability of high resolution LiDAR terrain data to refine the input interface locations represents another potential avenue.

A number of challenges were encountered during the development process. Consequently, the final dataset is associated with some limitations. These limitations could motivate further improvements in 3D geological modelling algorithms in general, or the present model in particular. They are as follows:

- The polarity (normal/overturned) of some surface orientation points in the frontal zone was not clear. In the horizontal plane, these points were located between vertical cross-sections. Given the extreme folding in the region, their polarities could not be easily confirmed. These points were omitted.
- The subsurface orientation data derived from the vertical cross-sections were taken as if there is no plunge (i.e. as if the cross-sections are perfectly orthogonal to the main fold). In reality, the plunge angle is spatially variable, and the true dip and dip direction at any given location are dependent on the plunge angle and as well as the geometry of any secondary folds in those locations. Making the assumption of orthogonality was thus the only practical way in which subsurface orientations could be assigned, which in turn was important for successfully modelling this structurally complex region. Given its intended application(s), this assumption is not expected to have a major impact on the model.
- In addition to the data prescribed, the implicit approach taken by GeoModeller inherently considers “geological concepts” when constraining the output. Whilst some of these constrains are highly beneficial (e.g. overlapping or “leaking” geological layers are not permitted), others, such as a tendency towards regularity in formation thickness throughout the domain, were more troublesome. This is because the thickness of some formations, most notably “i6_8”, does change drastically over short distances in our study area. Consequently, some interpolation artefacts such as disconnected “spheres” are present in areas of the model where the gradient in the potential field changes rapidly, particularly near the apices of sharp folds. In such a structurally complex environment, and with a large observation dataset, slightly improved results could have perhaps been obtained by assigning a greater weight to our observations and giving less freedom to the geostatistical interpolation.
- When computing the model, the same range value was used for both the interface data and the orientation data. In GeoModeller, it is not possible to specify a different nugget parameter for, say, the interface data according to whether it was derived from the surface maps or the vertical cross-sections. In other words, it is only possible to make a distinction between the interface and orientation data when assigning range and nugget effect values, which are global parameters. This was unfortunate in our case because, being directly observable, the surface data points are much less uncertain than subsurface contacts, which are highly dependent on interpretation. Hence, all observations of a certain type carried an equal weight in our model.
- The Quaternary cover is not represented by the model. The only available data pertaining to the unconsolidated Quaternary deposits are a series of shallow boreholes near the source of Le Rippaz, and reveal it to be discontinuous and heterogeneous. The Quaternary fill in the Vallon de Nant itself is likely to be a complex mixture of material having glacial, fluvial, and mass-movement origins. Reconstructing Quaternary cover in such environments is challenging in its own right. However, it is likely to be necessary for reliable hydrogeological flow simulations. As such, following geophysical surveys and their interpretation, plausible realisations of the heterogeneous structures could be generated using stochastic geostatistical simulation approaches such as Multiple Point Statistics^38^. An update of the present (bedrock only) model could potentially then be issued.

In conclusion, no 3D geological model the study region – which now forms the focus of a concerted interdisciplinary research effort – had been published or otherwise made available at the outset of our work. Whilst the development of a model with the requisite characteristics for hydrogeological and other applications was both technically challenging and time consuming, this data gap has now been addressed. The modelled formation interfaces correspond well with the location of these interfaces according to the input data, both at the surface and on available vertical cross-sections. Assuming the geological interpretation presented in the original maps and cross-sections is reasonable, the close matches obtained provide confidence that modelled representation of the geology also acceptable; a view that was upheld by additional comparisons with interpretive sketches.

In the sense that no new primary data has been collected, this work could be considered a data augmentation exercise. Our model thus demonstrates the considerable potential that exists to add value to existing geological data. We argue that it also amounts to more than the sum of its inputs; via the combination of the various input datasets and the process of geostatistical interpolation, it provides insight over the entire domain, ultimately forming an unprecedented geometrical description of the geological formations of the western and northern section of the Nappe de Morcles.

In contrast to certain previous publications which describe the development of 3D geological models only superficially, here, a detailed, step-by-step process has been presented. This should assist future researchers and practitioners in developing complex 3D geological models in future. Again in contrast to most previous instances, the data generated here are made freely-available, and care has been taken to ensure that they can be visualised in open source software. A range of applications across the earth and environmental sciences are likely to benefit if such work is conducted more consistently.

In one ongoing application, the geological model is being employed alongside various other datasets to parameterise physically-based, numerical simulations that integrate snow cover dynamics, surface-water flow, groundwater flow, and evapotranspiration. These simulations intend to elucidate the overall response of mountain hydrological systems to ongoing climatic change.

Finally, having been developed with the stringent, specific requirements of hydrological applications in mind, the model should also be suitable for a range of other applications, including rockfall hazard modelling, sediment provenance identification, hydro-chemical data interpretation, and pedogenic studies.

## Usage Notes

### Instructions to visualise the model data

To visualise the surfaces in ParaView (as in Fig. 3):

1. Download the software from https://www.paraview.org/, and install it.
2. Under “File”, select “Load State”
3. Navigate to “Geological_Model_State_File.pvsm”
4. Under “Load State Options”, select “Search file names under specified directory”, and ensure the directory path is correct.
5. Individual layers can be made transparent/non transparent by clicking on the “eye” symbol on the left hand side.

**Figure 9:**
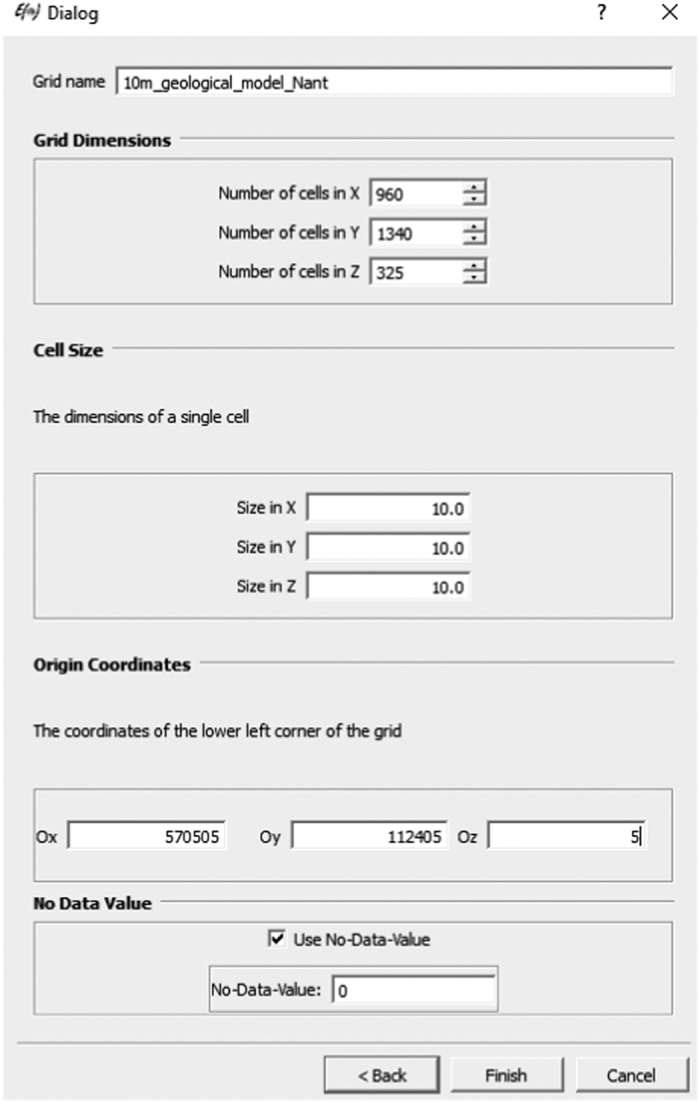
Parameters that should be entered in order to visualise the voxel data in SGeMS.

To visualise the voxel data in SGeMS:

1. Download the software from http://sgems.sourceforge.net/, and install it.
2. Select “Load Object”, navigate to the file “10 m.SGEMS” and click “Open”.
3. In the “Select object type” dialogue box, choose “cartesian grid”, and click “Next”.
4. Provide a name for the grid, and enter the values shown in the following screenshot (Fig. 9), then click “Finish”.
5. Once the data are loaded (which may take a few minutes), it may be viewed by checking the two tick boxes under the object tab.
6. The colour map can be changed if desired using the options under the “Preferences” tab. Also under this tab, by checking “Use Volume Explorer” and “Hide Volume”, a number of slices in the X, Y and Z planes can be visualised using the sliders.

Despite having specified the No-Data-Value on import, it does not seem possible make the grey area transparent. Nor does it seem possible to match the colour scheme in SGeMS to that used in this paper. An illustration of the model visualised in SGeMS is given above (Supplementary Figure 4).

To work with the data in GeoModeller (licence required):

1. Launch GeoModeller
2. Open the project by navigating to the “.xml” file provided.

## Additional information

**How to cite this article**: Thornton, J.M. *et al*. A 3D geological model of a structurally complex Alpine region as a basis for interdisciplinary research. *Sci. Data*. 5:180238 doi: 10.1038/sdata.2018.238 (2018).

**Publisher’s note**: Springer Nature remains neutral with regard to jurisdictional claims in published maps and institutional affiliations.

## Supplementary Material



Supplementary Figures

## Figures and Tables

**Table 1:** Datasets constituting the inputs to the development of the geological model.

**Type**	**Name**	**Author / owner**	**Key characteristics**	**Format**
Digital Terrain Model (DTM)	swissALTI^3D^	swisstopo	2 m native spatial resolution, vertical uncertainty ± 0.5 m < 2000m, 1-3 m > 2000m	Raster (.asc)
Surface geological maps	GeoCover (Sheet 58 “Morcles”)	swisstopo	1:25,000, shows formation interfaces, orientations (dip and dip direction) and faults	Vector (.shp) (pre-digitised from paper maps)
	GeoCover (Sheet 88 “Les Diablerets”)	swisstopo	1:25,000, shows formation interfaces, orientations (dip and dip direction) and faults	Vector (.shp) (pre-digitised from paper maps)
Vertical geological cross-sections	Explanatory booklet of Sheet 58 “Morcles”	Badoux (1971) (swisstopo)	Series of “stacked” cross-sections showing interpreted formation interfaces (start and end points not georeferenced)	Images in .pdf document
	Explanatory booklet of Sheet 88 “Les Diablerets”	Badoux and Gabus (1991) (swisstopo)	Series of “stacked” cross-sections showing interpreted formation interfaces (start and end points georeferenced)	Images in .pdf document
The lack of anthropogenic exploitation of the remote and protected study area means that no boreholes extending to the bedrock exist.				

**Table 2:** Association between numerical codes necessary for visualisation in SGeMS, the lithological formation codes in the model, and their descriptions (translated from the original French).

**Code**	**Formation**	**Description (Stage)**
0	“Out” / air	Above topographic surface
10	Basement	Crystalline massifs
11	a	Dark clayey shales (Aalenian)
12	i1i	Alternating marly shales and siliceous limestones (Lower Bajocian)
13	i1s	Siliceous limestones (Upper Bajocian)
14	i2	Limestones and dark shale (Bathonian)
15	i3_4	Shaley marls (Callovo-Oxfordian)
16	i5	Grey bedded limestones (Argovian)
17	i6_8	Compact limestones (Upper Malm)
18	i8c2	Alternating marls and clayey limestones (Upper Portlandian to Valanginian)
19	c2k	Predominantly dense limestones (Valanginian)
20	c3	Siliceous limestones (Hauterivian)
21	c4	Alternating marls and limestones (Lower Barremian)
22	c4_5	Light-coloured, dense limestones (Urgonian)
23	c6_8	Sandy shales and limestones (Upper Albian – Cenomanian)
24	e5	Layers of “Roc Champion” (Auversian)
25	e6c	Layers with Vivipares and Ceriths (Priabonian)
26	e6k	Limestone with small Nummulites (Priabonian)
27	e6o1	Parautochthonous Flysch (Upper Eocene to Lower Oligocene)
