# Publisher Correction: A 3D geological model of a structurally complex Alpine region as a basis for interdisciplinary research

**DOI:** 10.1038/s41597-019-0298-9

**Published:** 2019-12-13

**Authors:** James M. Thornton, Gregoire Mariethoz, Philip Brunner

**Affiliations:** 1 Centre for Hydrogeology and Geothermics, University of Neuchâtel, Rue Emile-Argand 11, 2000 Neuchâtel, Switzerland; 2 Institute of Earth Surface Dynamics, University of Lausanne, 1015 Lausanne, Switzerland

Correction to: *Scientific Data* 10.1038/sdata.2018.238, published online 30 October 2018

During typesetting, three paragraphs of text were erroneously removed from the Methods section of this Data Descriptor. These paragraphs have been re-inserted into both the HTML and PDF versions.

