# Supplementary material for: A 3D geological model of a structurally complex Alpine region as a basis for interdisciplinary research
Source: Sci Data. 2018 Oct 30;5:180238. doi: 10.1038/sdata.2018.238 (PMC6207069; doi:10.1038/sdata.2018.238)
Supplement: Supplementary Figures [file sdata2018238-s2.docx]

**Supplementary File 1**

Contents

| Supplementary Figure 1 | Sketch of the stratigraphy in the region of the Vallon de Nant | p. 2 |
| --- | --- | --- |
| Supplementary Figure 2 | Topographic surface of the geological model | p. 3 |
| Supplementary Figure 3 | Comparison between a sketch of the eastern side of the Vallon de Nant and a similar view within the modelled environment | p. 4 |
| Supplementary Figure 4 | Voxel model output (10 m resolution) visualised in SGeMs | p. 5 |

**
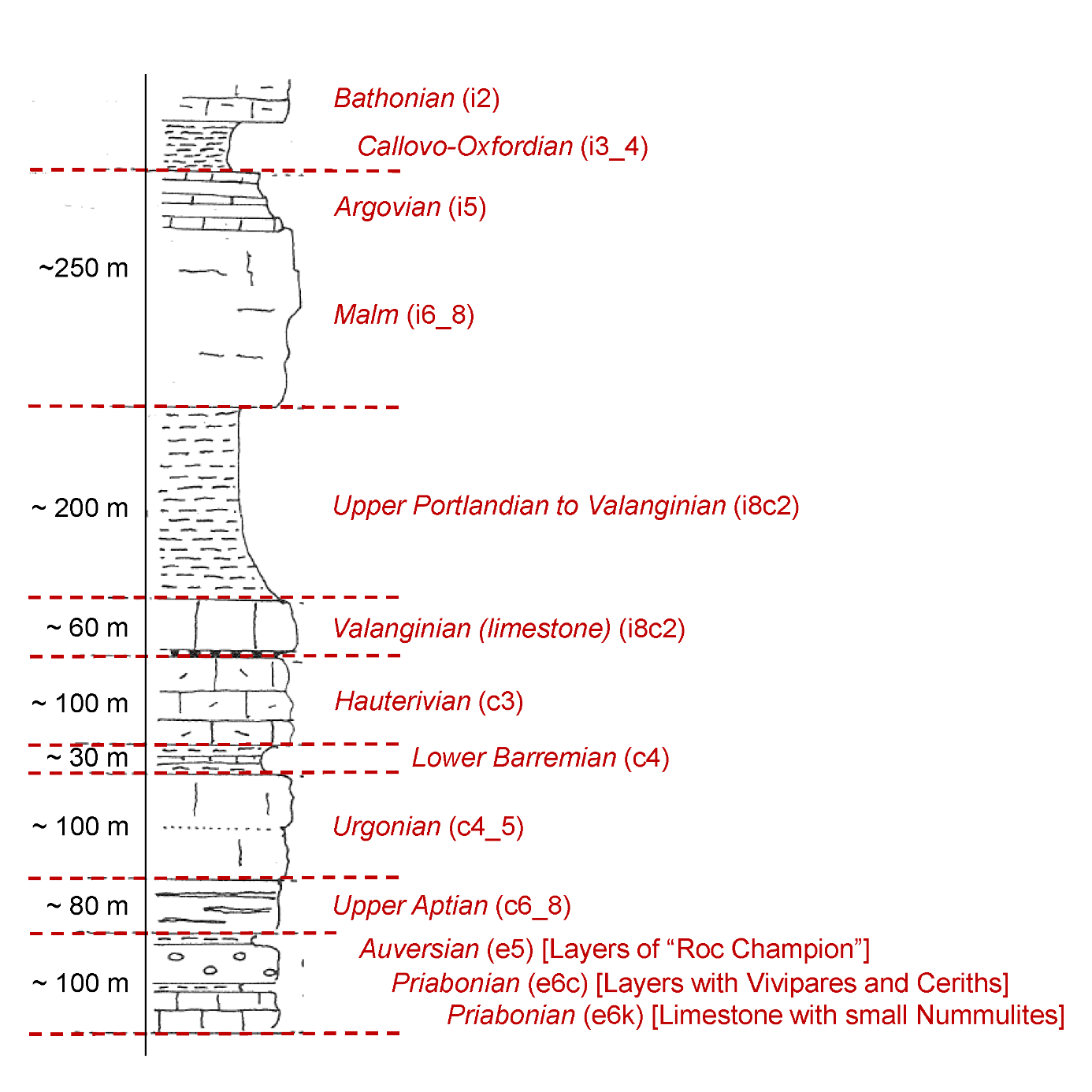
Supplementary Figure 1.** **Sketch of the stratigraphy in the region of the Vallon de Nant.** This figure is modified after Badoux^59^. Note that the sequence is represented as it is observed in this section of the nappe, i.e. is inverted, with the oldest formations above the younger ones.


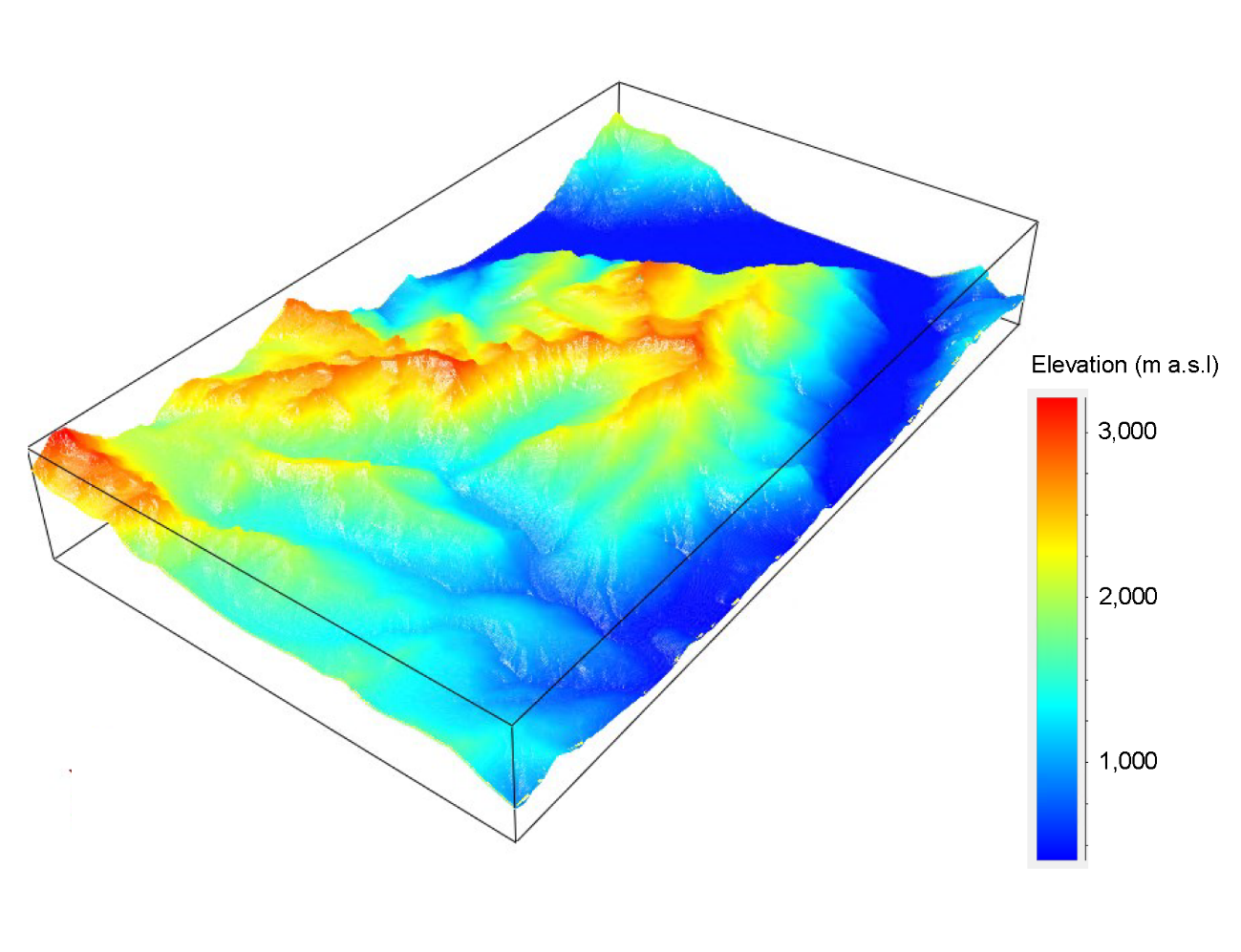


**Supplementary Figure 2. Topographic surface of the geological model.** This surface was defined according to the resampled incoming Digital Elevation Model (DTM) (10 m resolution), and visualised in the GeoModeller interface. Here, the view is towards the southeast.


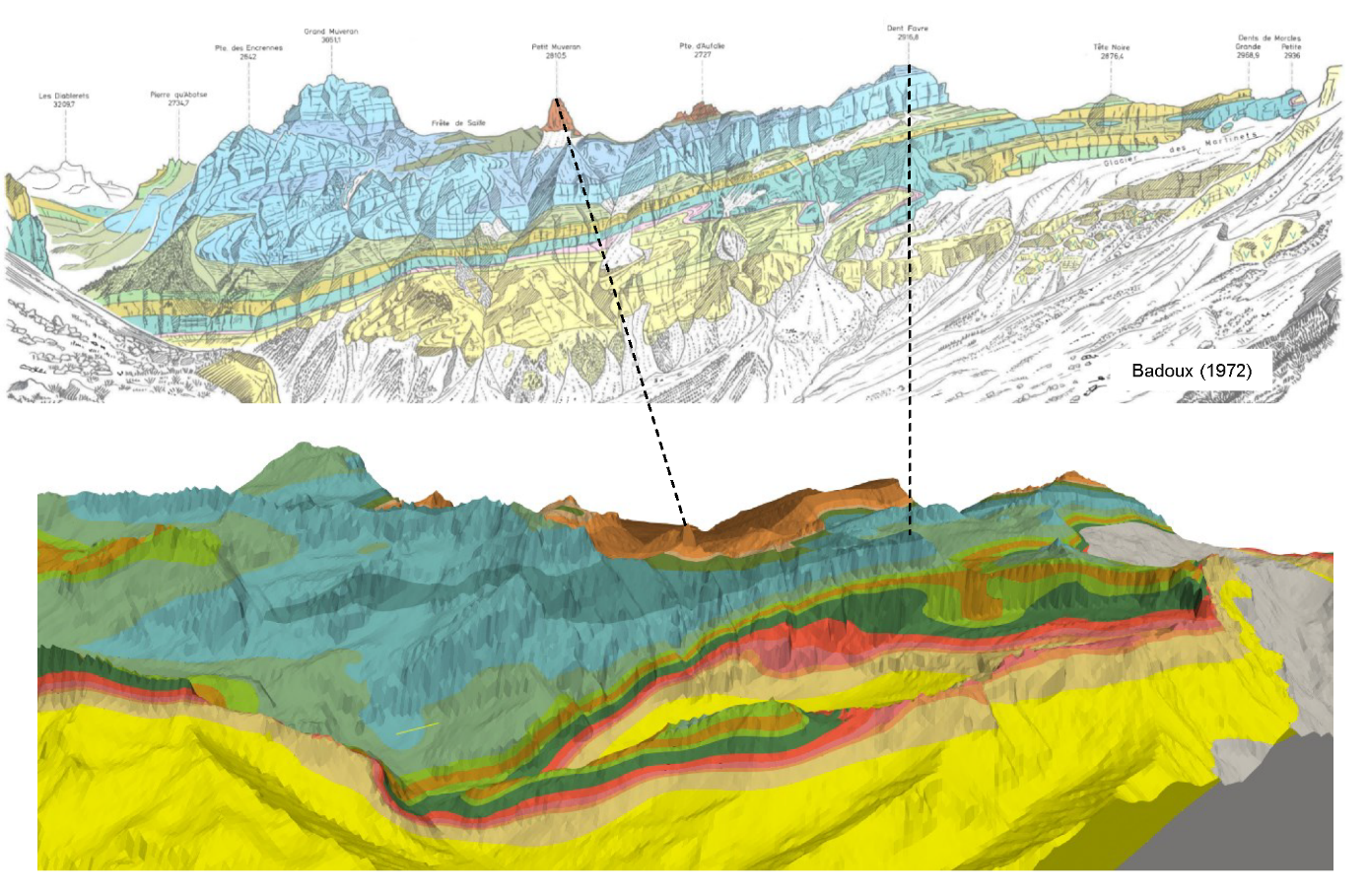


**Supplementary Figure 3. Comparison between a sketch of the eastern side of the Vallon de Nant and a similar view within the modelled environment.** The upper illustration was produced by Badoux^54^, whilst the lower was generated by the authors in Paraview.


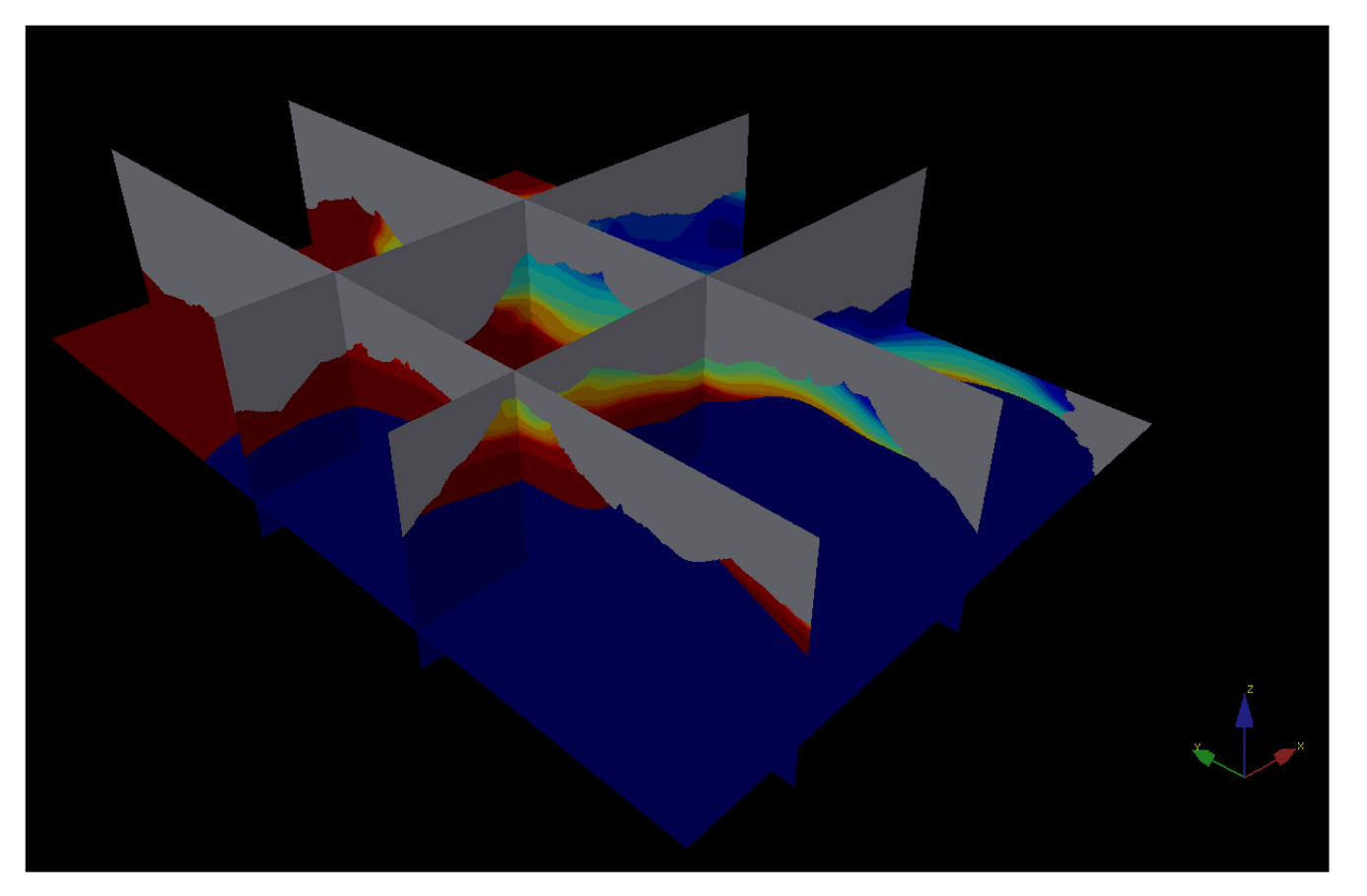


**Supplementary Figure 4. Voxel model output (10 m resolution) visualised in SGeMS.** Here, the view is towards the northeast.
